# Microbial viability and nutritional content of water kefir grains under different storage conditions

**DOI:** 10.1002/fsn3.4074

**Published:** 2024-03-05

**Authors:** Çağlar Gökırmaklı, Gülçin Şatır, Zeynep Banu Guzel‐Seydim

**Affiliations:** ^1^ Department of Food Engineering Suleyman Demirel University Isparta Turkey; ^2^ Department of Nutrition and Dietetics Suleyman Demirel University Isparta Turkey

**Keywords:** lactic acid bacteria, storage, sugary kefir, tibicos, vitamin, water kefir grains

## Abstract

Water kefir grains are an important source of probiotics, mainly containing lactic acid bacteria and yeasts. The aim of this study is to investigate the changes in microbial and chemical properties of water kefir grains during 1‐month storage at +4°C and −18°C. The initial content of lactobacilli, lactococci, and yeast in water kefir grains was 6.06, 6.33, and 5.93 log CFU/g, respectively. The number of lactobacilli, *Lactobacillus acidophilus*, and *Bifidobacterium* spp. in the water kefir grains were comparable, with slight changes at the end of refrigerated storage (*p* > .05). Lactococci and yeasts decreased significantly after both storage conditions compared to the initial content (*p* < .05). The dry matter and ash contents remained unchanged during storage (*p* > .05). Water kefir grains contained significant amounts of calcium, vitamin B_2_, vitamin B_6_, vitamin B_7_, and vitamin B_12_. Storage at both +4°C and −18°C did not affect the mineral and vitamin contents, except for Cu and Vitamin B_2_. The results indicate that the water kefir grains remained viable after storage at both temperatures. If water kefir grains need to be stored, it is recommended to store them at +4°C in sugared water as it ensures better survivability of the microbiota of the grains.

## Graphical abstract

Water kefir grains are abundant in vitamin B, especially vitamin B_7_. The total number of microorganisms decreased after storage at either +4°C or −18°C. Storage of the water kefir grains at +4°C is a better option for the viability of the microbial populations from the grains.

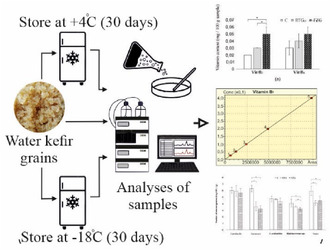

## 1 INTRODUCTION

Water kefir (WK) is a type of kefir that differs from milk kefir in terms of its chemical structure, physical appearance, and microbial content (Gökırmaklı & Güzel‐Seydim, 2022). It is popular in South America, Eastern Europe, and Russia (Lynch et al., 2021). Water kefir is produced by fermenting a carbohydrate‐containing solution with water kefir grains (WKG) at 21–25°C for 48–96 h (Pendón et al., 2022). Various substrates could be used to obtain WK beverages, such as apple, mandarin, persimmon, quince, grape, kiwi, coconut, sugarcane juice, honey, grape molasses, ginger, and onion (Cufaoglu & Erdinc, 2023; Güzel‐Seydim et al., 2023; Lynch et al., 2021; Moretti et al., 2022; Pendón et al., 2022). In addition to the fermentation environment, various fermentation parameters related to WK production have been studied (Gökırmaklı et al., 2023; Laureys et al., 2018, 2019, 2021; Laureys & De Vuyst, 2014, 2017). According to the results of these studies, changes in grain inoculum ratio, grain source and microbiota, temperature, oxygen level, nutritional quality of fermentation substrate, and fermentation time affect the final fermented product of WK.

WKG typically contains a variety of lactic acid bacteria and yeasts, including members of the Firmicutes group such as *Lactobacillus* spp., *Streptococcus* spp., and *Lactococcus* spp., as well as acetic acid bacteria and yeasts like *Saccharomyces* spp. (Patel et al., 2022; Yerlikaya et al., 2022). Some of the microorganism groups in WKG exhibited probiotic properties (Angelescu et al., 2019; Koh et al., 2018; Romero‐Luna et al., 2019, 2020). Previous studies have investigated the health impacts of consuming WK (Cufaoglu & Erdinc, 2023; Moretti et al., 2022). Several studies have shown that consumption of WK may have potentially favorable effects against irritable bowel syndrome (Gökırmaklı et al., 2023) and has gastro‐protective (Brasil et al., 2019), cholesterol‐lowering (Rocha‐Gomes et al., 2018), antidiabetic and antihyperlipidemic (Alsayadi et al., 2014), antimicrobial (Gonda et al., 2019), anti‐inflammatory (Hsieh et al., 2012), antioxidant (Alsayadi et al., 2013), and hepato‐protective properties. However, the ash and protein contents of WK grains are lower than those of well‐known milk kefir. Nevertheless, WK grains are a good source of essential minerals such as potassium, calcium, sodium, magnesium, iron, and copper (Gökırmaklı & Güzel‐Seydim, 2022).

Currently, there is a growing demand among vegan consumers for probiotic beverages like WK (Dahal et al., 2020; Moretti et al., 2022; Mousavi et al., 2023). However, the available information on storing water kefir grains was limited. Only one study, conducted by Laureys et al. (2017), concluded that freezing and thawing of WKG leads to irreversible damage. However, no comprehensive study has been conducted on how the storage of WKG affects its chemical and microbial properties. The aim of this study is to investigate the changes in microbial and chemical properties of WKG when stored at +4°C and −18°C for 30 days.

## 2 MATERIALS AND METHODS

### 2.1 Chemicals and materials

All chemicals used for chromatographic analyses, including vitamin and mineral analyses, were of chromatographic purity. The chemicals and microbial culture media were supplied by Sigma Aldrich Inc. (St. Louis, MO, USA). White crystal sugar (https://torku.com.tr/torku‐kristal‐seker‐polietilen‐ambalaj) was used as a carbohydrate source. Natural spring water (https://www.hayatsu.com.tr/) was used as the water source.

### 2.2 Water kefir grains

WKG were kindly provided by Danem Inc. (https://www.kefirdanem.com/) and a household in Antalya, Turkey. A total of 1200 g of water kefir grains were obtained from each source. The grains were then divided equally for each experimental group, for example, 600 g for each storage condition. Finally, each group of grains was divided equally to provide two replications and four parallels for a correct experimental design. Each grain was used individually without being mixed with grains from the other origin. Two different origins were preferred to prevent any bias. This is because the properties of water kefir grains could be affected by their origin. All of WKG were activated prior to use for experimental study. To ensure proper activation of the WKG before starting the experiments, they were fermented in a sterile 5% (w/v) white sugar solution at 25°C for 24 h. The WKG were then filtered through a sterile sieve and treated with sterile distilled water at 25°C. Subsequently, the kefir grains were divided into two groups. One group was stored in sterile 5% (w/v) sugared water at +4°C for 30 days (RTG), while the other group was stored in sterile 5% (w/v) sugared water at −18°C for 30 days (FZG). The kefir grains were analyzed at the beginning (day 0) and at the end of the storage period (day 30). During the analysis, kefir grains were defrosted at room temperature (25°C) without any intervention. During the thawing process, the ambient temperature was not changed and all samples were thawed in a relatively short time interval of about 15 min.

### 2.3 Microbiological analysis

Microbiological content analysis was performed based on Mossel et al. (1995), Shah (2000), and Roy (2001). Briefly, 2 g of WKG was mixed with 18 mL of sterile peptone water in a stomacher (Interscience‐BagMixer 400, St Nom, France) and ground for 3 min. Subsequently, 1 mL was taken and serially diluted. Then, de Man, Rogosa, and Sharpe (MRS) agar was used for lactobacilli, M17 agar was used for lactococci, MRS agar with 10% (w/v) sorbitol was used for *Lactobacillus acidophilus*, MRS agar containing appropriate concentrations of neomycin, nalidixic acid, lithium chloride, and paromomycin sulfate as described by Roy (2001) was used for *Bifidobacterium* spp. and potato dextrose agar contained 0.14% lactic acid was used for yeast count. On the other hand, all grains were tested for the possible presence of pathogens on violet red bile (VRB) agar. All petri dishes except yeast were incubated at 37°C under anaerobic conditions (6% CO_2_) for 48 h. The yeasts were incubated under normal atmospheric conditions. No special air modification was used. For yeast enumeration, petri dishes were incubated at 25°C for 120 h.

### 2.4 Proximate composition analysis

A Shimadzu MOC63U moisture analyzer (Kyoto, Japan) was used to determine the dry matter content of WKG. For this purpose, 3.0 ± 0.05 g of sample was used and the results obtained were expressed as percentage dry matter content. The ash content of WKG was analyzed according to the procedure of AOAC Official Method 900.02 (AOAC, 2005). Each sample (3.0 ± 0.05 g) was taken for combustion in a muffle furnace (Nuve MF 120, Ankara, Turkey) at 250°C for 1 h and at 550°C for 6 h.

### 2.5 Mineral content analyses

Mineral content analyses were conducted following the method of Gökırmaklı and Güzel‐Seydim (2022). Briefly, a 0.3 g of sample was treated with 8 mL of nitric acid (65% v/v) and 2 mL of hydrogen peroxide (30% v/v) using the wet composition method. The sample was then diluted to a final volume of 20 mL with distilled, deionized water. An inductively coupled plasma optical emission spectrometer (ICP‐OES, Perkin Elmer OPTIMA 5300 DV, Shelton CT, USA) was used for the measurement. The working conditions of the ICP‐OES device are presented in Supplementary Table S1.

### 2.6 Vitamin analysis

#### 2.6.1 Determination of vitamins B_1_ , B_2_ , B_3_ , and B_6_

Thiamine, riboflavin, niacin, and pyridoxine were detected using the method previously applied by Satir and Guzel‐Seydim (2016). The method involved extraction of the vitamins, followed by acid treatment for partition, and quantification by HPLC (Shimadzu, LC‐20AD, Kyoto, Japan). Further details on the HPLC conditions can be found in the supplementary file S1 (Supplementary S1A and S1B).

#### 2.6.2 Determination of vitamins B_7_ and B_12_

A buffer solution of C_2_H_3_NaO_2_ in the presence of NaCN at 100°C for 30 min was utilized to extract biotin and cobalamin. After the purification of extracts, they concentrated with immunoaffinity column (Table S2). Vitamins were quantified via HPLC (Shimadzu, LC‐20 AD, Kyoto, Japan). Ultraviolet detection wavelengths for biotin and cobalamin were 200 nm and 361 nm, respectively (Eitenmiller et al., 2016). The supplementary file S1 (Supplementary S1A and S1B) provides details regarding HPLC conditions.

#### 2.6.3 Determination of vitamin C

The vitamin C in WKG was determined via reverse‐phase HPLC (Shimadzu, LC‐20AD, Kyoto, Japan) (Satir & Guzel‐Seydim, 2016). The supplementary file S1 (Supplementary S1A and S1B) provided details regarding HPLC conditions. HPLC operating conditions for all vitamin analyses are presented in Table S2.

### 2.7 Statistical analysis

In this study, two different water kefir grains with different origins were used. For each type of grain of different origins, two replicates and two parallels were measured. In total, for each origin of grain, two replications and four parallel values were obtained. The results were expressed as mean ± standard deviation. For statistical analysis of the results, a one‐way analysis of variance (ANOVA) test was performed using the IBM SPSS v. 22.0 program (SPSS Inc., Chicago, USA). Tukey's *b* test was preferred to determine the significance between the results of the experiments (*p* < .05) for all analyses. For the statistical analysis, the results of the storage conditions at +4°C and −18°C were compared.

## 3 RESULTS AND DISCUSSION

**FIGURE 1:**
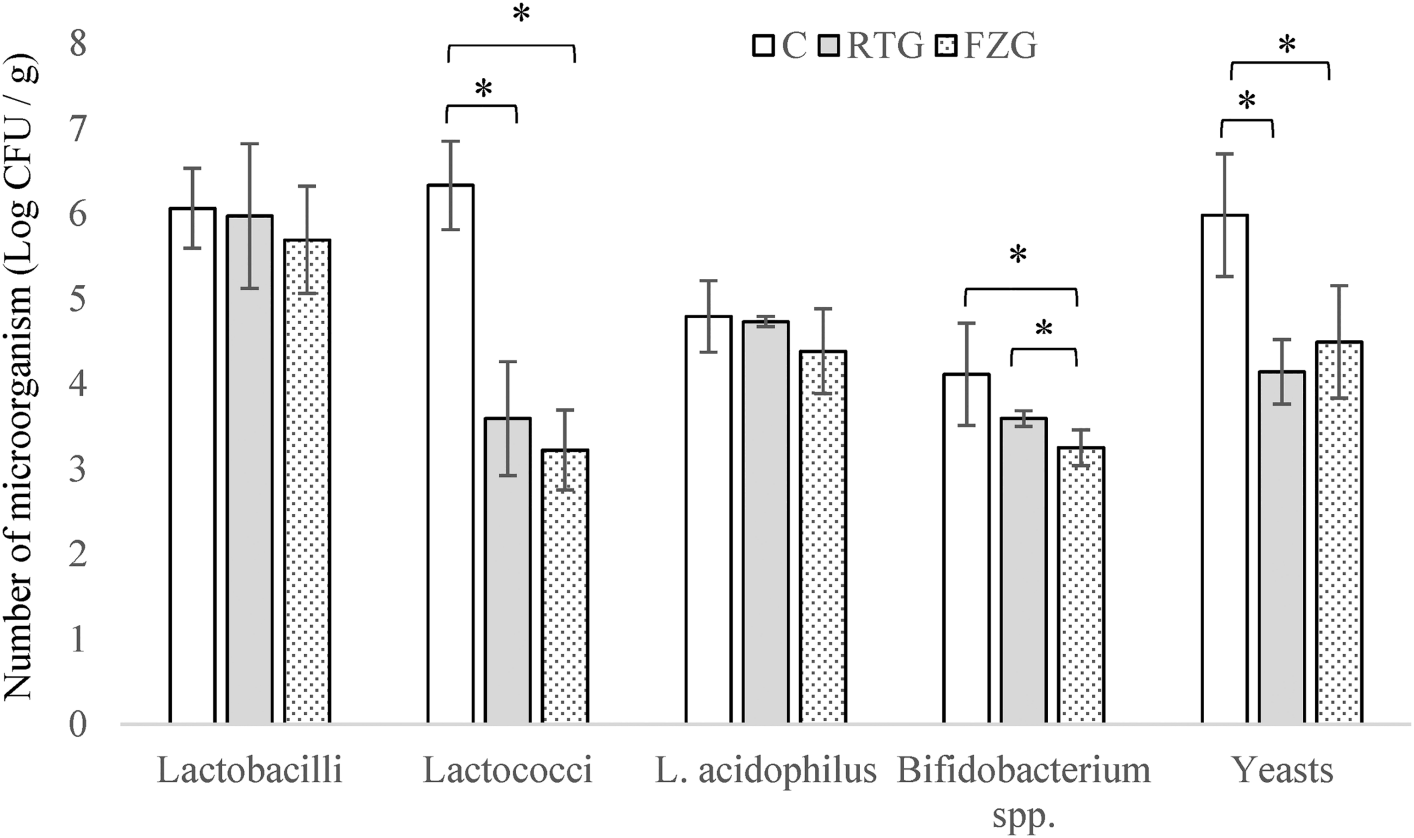
Microbiological contents of water kefir grains in different storage conditions. The sign “*” indicates differences between groups. C: Control, RTG: Water kefir grains stored at 4°C for 30 days, FZG: Water kefir grains stored at −18°C for 30 days.

Based on the VRB agar test results, no pathogenic contamination was found in the grains. The study was conducted under sterile conditions and all WKG were treated according to appropriate microbial protocols. Initially, the WKG had a high microbial content. However, after 30 days of storage in both the RTG and FZG groups, the number of microorganisms decreased. The lactobacilli and *L. acidophilus* contents in the WKG were similar, with slight differences at 0 and 30 days (*p* > .05). Regarding their initial levels, significant decreases were noted for lactococci, *Bifidobacterium* spp., and yeasts (*p* < .05). The abundance ratio of lactobacilli increased, while that of lactococci decreased under both storage conditions. The abundance ratio of other microbial groups varied depending on the storage temperature (Figure 1). The numbers of lactobacilli and lactococci were 6.06 and 6.33 log CFU/g, respectively, as shown in Figure 1. The numbers of *L. acidophilus* and *Bifidobacterium* spp. were detected as 4.79 and 4.11 log CFU/g, respectively, as shown in Figure 1.

WKG microbiota is mainly composed of lactic acid bacteria and yeasts (Laureys & De Vuyst, 2014). Maintenance of kefir grains is critical for preserving a well‐balanced microbiota in the final product (Laureys et al., 2018). The lactic acid bacteria and yeast ratio are critical in kefir grains (Laureys et al., 2018). The WKG sample contained between 6.04 and 9.18 log CFU/mL of lactobacilli. Yeasts were reported as 5.54–8.30 log CFU/mL in previous studies (da Miguel et al., 2011; Gökırmaklı & Güzel‐Seydim, 2022). The results obtained were in line with those of previous studies. Previous studies have found similar trends for bacteria during food storage periods. For instance, Grønnevik et al. (2011) reported a significant decrease of 3 log CFU/mL in the number of lactococci after kefir had been stored for 4 weeks. Chou and Hou (2000) observed that the number of *Bifidobacterium infantis* and *B. longum* in the fermented beverage decreased by 0.44 and 3.18 log CFU/mL, respectively, after storage at 5°C for 10 days. *Bifidobacteria* species are susceptible to aerobic and acidic conditions, which are common in most food products.

Both storage conditions had a similar effect on the microbial content of WKG at the end of storage (*p* > .05). The yeast content of WKG decreased significantly during storage regardless of storage temperature (*p* < .05). WKG stored at 4°C contained significantly more *Bifidobacterium* spp. than WKG stored at −18°C for 30 days (*p* < .05). After storage, the WKG contained lactobacilli, lactococci, *L. acidophilus*, *Bifidobacterium* spp., and yeast, although in lower amounts, for both storage conditions.

At day 0, WKG had a dry matter content of 13.38% and an ash content of 0.04% (Table 1). After 30 days of storage at +4°C or −18°C, there was no significant effect on the dry matter and ash content of the grains (*p* > .05) (Table 1). According to Gökırmaklı and Güzel‐Seydim (2022), the dry matter and ash contents of WKG were 17.50% and 0.06%, respectively. Coma et al. (2019) reported a dry matter content of 14% for WKG, while Pidoux et al. (1988) reported a dry matter content of 11.5%. In addition, according to Pidoux et al. (1988), the dry matter of WKG included mostly polysaccharides. Our findings were compatible with previous results.

**TABLE 1:** Dry matter (%) and ash contents (%) of water kefir grains in different storage conditions.

	Dry matter (%)	Ash content (%)
C	13.38 ± 0.92^a^	0.04 ± 0.03^a^
RTG	11.32 ± 2.49^a^	0.07 ± 0.03^a^
FZG	11.90 ± 3.33^a^	0.06 ± 0.02^a^

*Note*: Results expressed as mean ± standard deviation. Different lower case letters in the same column indicate statistically significant differences between samples (*p* < .05). C: Control, RTG: Water kefir grains stored at 4°C for 30 days, FZG: Water kefir grains stored at −18°C for 30 days.

WKG contained 0.100 mg/g of calcium, 0.010 mg/g of copper, 0.030 mg/g of sodium, and less than 0.001 mg/g of magnesium (Table 2). These minerals have important functions in the human body. For instance, calcium promotes healthy bones and a healthy immune system, and regulates blood pressure. Copper improves the healing process in the human body, while sodium has many crucial functions, such as maintaining normal heart function and providing electrolyte stability. Magnesium is essential for creating protein, maintaining a healthy immune system, and transmitting nerve signals (Gharibzahedi & Jafari, 2017). All these functional properties are vital to the maintenance of normal functions of human body. For this reason, the recommended daily intake of minerals has been studied and some limits have been postulated by various scientists (Gharibzahedi & Jafari, 2017). These minerals are also crucial for the stability of the grain structure and the continuity of biomass production of the water kefir grain.

**TABLE 2:** The mineral content of water kefir grains (mg/g) in different storage conditions.

	Ca 317.933	Cu 327.393	Mg 285.213	Na 589.592
Control, *T* = 0	0.120 ± 0.020^a^	0.010 ± 0.004^a^	<0.001	0.030 ± 0.010^a^
RTG, *T* = 30	0.100 ± 0.020^a^	0.010 ± 0.001^a^	<0.001	0.040 ± 0.004^a^
FZG, *T* = 30	0.097 ± 0.001^a^	0.003 ± 0.001^b^	<0.001	0.034 ± 0.001^a^

*Note*: Results expressed as mean ± standard deviation. Different lower case letters in the same column indicate statistically significant differences between samples (*p* < .05).

Storage of the WKG at +4°C or −18°C for 30 days resulted in almost no significant change in mineral content, except Cu content. One possible explanation for this situation is the differences between the adsorption speed of minerals from the environment depending on the pH of the environment and process conditions (e.g., freezing) (Volpi et al., 2019). The results were consistent with the ash and dry matter content (Table 1). In a previous study (Gökırmaklı & Güzel‐Seydim, 2022), the contents of Ca, Cu, Mg, and Na in WKG were reported as 0.28 ± 0.19, 0.088 ± 0.02, 0.040 ± 0.024, and 0.735 ± 0.366 mg/g, respectively.

**FIGURE 2:**
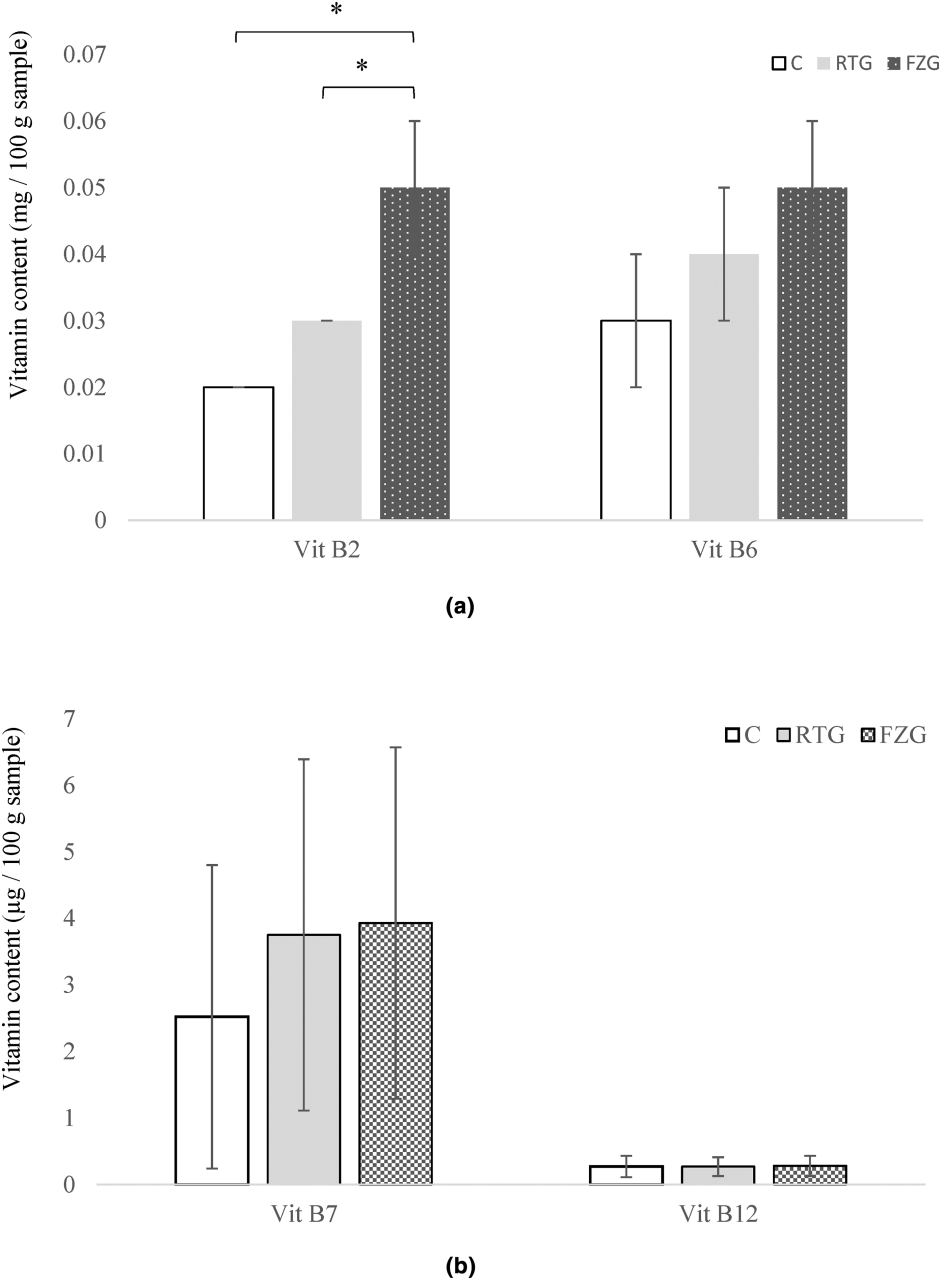
Vitamin contents of water kefir grains in different storage conditions. (a) Vitamin B_2_ and Vitamin B_6_, (b) Vitamin B_7_ and Vitamin B_12_. The sign “*” indicates differences between groups. The levels of vitamin B1, vitamin B3, and vitamin C were below the limit of detection (LOD) in all test groups. LOD for vitamin B1, vitamin B3, and vitamin C: 0.1 *μg* /100 g sample. C: Control, RTG: Water kefir grains stored at 4°C for 30 days, FZG: Water kefir grains stored at −18°C for 30 days.

The changes in vitamin contents of WKG stored at 4°C and −18°C are shown in Figure 2. WKG had vitamins B_2_, B_6_, and B_12_ (Figure 2).

WKG contains a relatively high amount of vitamin B_7_, which is crucial for energy metabolism (LeBlanc et al., 2011). In addition, sources of vitamin B_12_ are generally known to be animal foods (Satir, 2022). Therefore, it is crucial to know that WKG contain vitamin B_12_; probably due to microbial production of the specific microorganism(s). Some lactic acid bacteria can synthesize aqueous vitamins, especially B vitamins (LeBlanc et al., 2011). It would be necessary for vegans to have a plant‐based probiotic source with vitamin B_12_ content. On the other hand, vitamin B_2_ deficiency is the most widespread vitamin deficiency in developing countries (Mazzantini et al., 2022). The vitamin B_2_ content of WKG is valuable for overcoming this deficiency (Figure 2). The results indicate that vitamin B_2_ levels increased during the freezing process (Figure 2). B‐group vitamins are of water‐soluble vitamins. The freezing of WKG can prevent the transfer of vitamin B_2_ into the environment, for example, meltwater. Moreover, individually, some grains may contain higher amount of vitamin B_2_. As a result, vitamin B_2_ was seemed as increased under freeze storage conditions depending on these situations. Refrigerated and frozen storage of WKG did not decrease the water‐soluble vitamins (*p* > .05). It is important to note that water‐soluble vitamins in WKG were protected during storage. The levels of vitamins B_1_, B_3_, and C levels in WKG were detected below the detection limit.

Storage of WKG at 4°C or −18°C resulted in some microbial changes; however, the chemical content was not significantly affected. Laureys et al. (2017) reported that freezing and thawing WKG resulted in irreversible damage. Additionally, we observed that the WKG was relatively smaller after storage in frozen conditions, but storage of WKG at 4°C did not affect the grain sizes. On the other hand, the WKG remained viable at both storage temperatures regarding microbial and chemical component contents. It is concluded that the stored WKG are grown under suitable fermentation conditions with nutrient requirements, and they could grow; however, further studies are needed.

## 4 CONCLUSION

Daily maintenance is crucial for preserving the microbial composition and viability of WKG. Our results indicate that grain storage has an impact on microbial content, particularly lactococci and yeast. On the other hand, the chemical composition of WKG was relatively stable during storage. WKG had significant contents of calcium, vitamin B_2_, vitamin B_6_, vitamin B_7_, and vitamin B_12_. Storage at +4°C and −18°C did not affect the mineral and vitamin contents except Cu and Vitamin B_2_. When storage is necessary, it is recommended to store the WKG at +4°C in sugared water.

## AUTHOR CONTRIBUTIONS

**Çağlar Gökırmaklı:** Conceptualization (equal); data curation (equal); formal analysis (equal); methodology (equal); writing – original draft (equal); writing – review and editing (equal). **Gülçin Şatır:** Conceptualization (equal); data curation (equal); formal analysis (equal); methodology (equal); writing – original draft (equal); writing – review and editing (equal). **Zeynep Banu Guzel‐Seydim:** Conceptualization (equal); data curation (equal); formal analysis (equal); methodology (equal); supervision (equal); writing – original draft (equal); writing – review and editing (equal).

## CONFLICT OF INTEREST STATEMENT

None.

## ETHICS STATEMENT

This article does not contain any studies with human participants or animals performed by any of the authors.

## Supporting information


Data S1.

## Data Availability

The data that support the findings of this study are available from the corresponding author, Ç.G, upon a reasonable request.
