# Supplementary material for: Microbial viability and nutritional content of water kefir grains under different storage conditions
Source: Food Sci Nutr. 2024 Mar 5;12(6):4143–50. doi: 10.1002/fsn3.4074 (PMC11167166; doi:10.1002/fsn3.4074)
Supplement: Supplementary file 1 — Data S1. [file FSN3-12-4143-s001.docx]

**Supplementary Files**

**S1. Detailed Procedure of Water-Soluble Vitamins**

**S1A. Analytical methods**

**B_1_, B_2_, B_3_, B_6_**

10 g of homogenized water kefir grain sample was put in a 100 mL flask, then 60 mL 0.1 N hydrochloric acid solution was added and autoclaved at 121 °C for 30 min. After that, enzymatic treatment was performed to liberate the phosphorylated forms of thiamine (TMP, TDP, and TTP), riboflavin (FAD and FMN), and vitamin B6 (PLP, PNP, and PMP). There is no need for enzymatic extraction for niacin. For this reason, the sample is cooled, directly filtered, and injected into the HPLC.

*The extraction procedure for thiamine, riboflavin, and pyridoxine is as follows*: after cooling to room temperature, pH is adjusted to 4.5 using sodium acetate (2.5 mM) solution. 100 mg taka-diastase, 10 mg papain, 5 mg acid phosphatase, 5 mg β-glucosidase, and 10 mg α-amylase were added to the sample and incubated for 18 h for pyridoxine and 3 hours for thiamine and riboflavin at 37 °C in shaking water bath (GFL 1092, Burgwedel, Germany) with 150 rpm. Then, the samples were cooled to room temperature, and the volume was completed to 100 ml 0.1 N hydrochloric acid solution (for B_3_) and HPLC grade water (for B_1_, B_2_, and B_6_); afterward, it was filtered with the 0.45 μm filter and injected into the HPLC.

Due to its structure, thiamine can not be detected in the fluorescence detector alone. Therefore, pre-column derivatization is required to be detected by the fluorescence detector. Detection methods are described by Finglas et al. (1984); which was used with some modifications. 25 mL of the filtered sample is taken up in 50 ml polyethylene tubes, 1.5 mL of potassium ferricyanide solution (0.25 g of potassium ferricyanide dissolved in 25 mL of 15% sodium hydroxide solution) is added, adjusted to pH 7.0-7.1 with ortho-phosphoric acid and injected into HPLC after filtration through 0.45 μm filter. The mobile phase was prepared by mixing 750 mL of water and 250 mL of methanol, and 10 mM KH_2_PO_4_. The mixture was filtered through with 0.45 μm filter. Excitation and emission wavelengths were set at 366 and 445 nm for thiamine, respectively. An Agilent ZORBAXE Eclipse XDB-C18 column (4.6 × 150 mm, 5 μm, Agilent Technologies) was used, and a flow rate of 0.8 mL/min at 25 °C.

The riboflavin content in water kefir grain sample was determined by HPLC, consisting of a Shimadzu RF 20A pump with a Shimadzu RF-10AXL fluorescence detector (FLD) (Shimadzu Corporation, Kyoto, Japan). The mobile phase was prepared by mixing 750 ml of water and 250 ml of methanol. The mixture was filtered through with 0.45 μm filter. Excitation and emission wavelengths were set at 445 and 530 nm for riboflavin, respectively. An Agilent Eclipse XDB-C18 column (4.6 × 150 mm, 5 μm, Agilent Technologies) and a flow rate of 1 mL/min were used.

Similarly, as in thiamine, post-column derivatization is required to determine niacin. For post-column derivatization, a photochemical derivatization system was established. Detection methods described by Lahely et al. (1999) were used with some modifications. The derivatization system was made by wrapping a Teflon tubing with a length of 20 m and a diameter of 0.5 mm on a UV-A lamp 60 cm long. The system is connected between the analytical column and the fluorescence detector. The mobile phase should be prepared daily and protected from light. The mobile phase was prepared by mixing 50 mM KH_2_PO_4_, 7.5 mL of % 30 H_2_0_2_, and 1 ml of copper (II) sulfate solution dissolved in 100 mL of water, and then the mobile phase was filtered. The fluorescence detector was set to excitation wavelength of 325 nm and an emission wavelength of 480 nm, respectively. An Agilent LiChrosper 60 RP-select B (250 mm×4.0 mm, 5 µm), Agilent Technologies) and a flow rate of 1 mL/min were used.

The level of B_6_ vitamins (PL, PN, and PM) in samples was determined using a reversed-phase HPLC method. HPLC conditions described by Kall (2003) were used with some modifications. HPLC system was a Shimadzu LC 20AT pump with a Shimadzu RF-20A fluorescence detector (Shimadzu Corporation, Kyoto, Japan). The mobile phase was prepared as follows: the buffer solution was prepared by dissolving 11 g of KH_2_PO_4_ and 0.5 g of 1-octane sulfonic acid in 940 mL deionized water (50 mM). Then, pH was adjusted to 2.9 with ortho-phosphoric acid with at least volumes of acetonitrile and acetonitrile: water (95:5 v/v). The fluorescence detector excitation and emission wavelengths were set at 290 and 395 nm, respectively. B_6_ vitamins were separated with ACE 5 C18 (250 mm×4.6 mm, 5 µm) column (Agilent, USA) with a 1.0 mL/min flow rate. The column oven temperature was set to 30 °C.

**Vitamin B_7_**

5 g sample was accurately weighed into a 100 mL amber glass screw bottle. 50 mL of 0.1 M sodium phosphate buffer pH 7.2, 4 g pancreatin, and stirring for 10 min. 6 ml of sodium ascorbate 10% were added under agitation for 5 min. The solution was incubated at 37 °C for 2 hours in a shaking water bath with 150 rpm (GFL 1092, Burgwedel, Germany) and then incubated at 100 °C for 30 min in a second shaking water bath (GFL 1092, Burgwedel, Germany). After cooling at room temperature, the solution was quantitatively transferred into a 100 ml amber glass flask and filled to the mark with 0.1 M sodium phosphate buffer. The solution was centrifuged at 4000 rpm for 15 min and filtered. 20 ml of the extract was loaded onto an immunoaffinity column (6 mm × 8 mm i.d.; prototype, R-Biopharm, Saint Didier auMont d’Or France) previously washed with 20 mL 0.1 M phosphate buffer. The column was washed with 15 mL of water and dried by passing through the air with a syringe. The Vitamin B7 was then eluted with 3 mL of methanol. The eluate was concentrated to dryness under air at 80-90° C and then reconstituted in 1 ml solution A before the HPLC-UV analysis.

An aliquot (100 µL) of the samples was placed on an HPLC column (ACE 5 C18 (250 mm×4.6 mm, 5 µm) equilibrated with water: acetonitrile (875/125) (v/v) containing 250 μL trifluoroacetic acid. The corrinoids were isocratically eluted with the same solution at a flow rate of 0.8 mL/min at 40 °C and monitored by determining the absorbance at 200 nm.

**Vitamin B_12_**

15 g sample was accurately weighed into a flask. 50 mL of 50 mM sodium acetate buffer pH 4.0, 0.5 g α-amylase, and 2 g of pepsin were stirred for 10 min. 1 ml of sodium cyanide 1% and potassium cyanide 1% were added under agitation, and the solution was incubated at 37 °C for 30 min. The solution was incubated at 100 °C for 35 min in a second shaking water bath (GFL 1092, Burgwedel, Germany). After cooling at room temperature, the solution was quantitatively transferred into a volumetric 100 ml flask and filled to the mark with 50 mM sodium acetate buffer. 20 ml of the extract was loaded onto an immunoaffinity column (6 mm × 8 mm i.d.; prototype, R-Biopharm, Saint Didier auMont d’Or France) previously washed with 10 mL of distilled water. The column was then washed with 15 mL of water and dried by passing through the air with a syringe. The Vitamin B_12_ was then eluted with 3 mL of methanol. The eluate was concentrated to dryness under air at 60-70° C and then reconstituted in 300 μl solution A before the HPLC-UV analysis.

We loaded the supernatant onto an EASI-EXTRACT ® B_12_ Immunoaffinity Column (P80/P82) (R-Biopharm AG, Darmstadt, Germany) and then purified the B_12_ compounds according to the recommended protocol. An aliquot (100 µL) of the samples was placed on HPLC column (ACE 5 C18 (250 mm×4.6 mm, 5 µm) equilibrated with water: acetonitrile (875/125) (v/v) containing 250 μL trifluoroacetic acid. The corrinoids were isocratically eluted with the same solution at a flow rate of 0.8 mL/min at 40 °C and monitored by determining the absorbance at 361 nm.

**Vitamin C**

The extraction of Vitamin C was performed with some modifications to the previous methodologies of Satir and Guzel-Seydim, 2016. Firstly, 80 mL of meta-phosphoric acid solution (3%) was added to five grams of samples and the mixture was mixed in a shaker for 10 min. Using meta-phosphoric acid solution (3%), the volume was completed to 100 mL. Next, the final solution was filtered using a CA filter (0.45 μm). Lastly, the filtrate was transferred into HPLC vials before the HPLC analysis. The reverse-phase HPLC system comprised a Shimadzu LC 20AT pump and a Shimadzu SPD-20A UV/VIS detector (Shimadzu Corporation, Kyoto, Japan). The mobile phase preparation was achieved by 10 mM KH_2_PO_4_ in 1000 mL of distilled water and using ortho-phosphoric acid; the pH was adjusted to 2.8-2.9. ACE 5 C18 (250 mm×4.6 mm, 5 µm) (ACE, Scotland) was employed to separate related compounds. The flow rate was 0.5 mL/min. The wavelength of the detector was 254 nm.

**REFERENCES**

Finglas, P. M., Faulks, R. M. (1984). The HPLC analysis of thiamin and riboflavin in potatoes. *Food Chemistry*, 15(1), 37-44.

Kall, M. A. (2003). Determination of total vitamin B_6_ in foods by isocratic HPLC: a comparison with microbiological analysis. *Food Chemistry*, 82(2), 315-327.

Lahély, S., Bergaentzlé, M., Hasselmann, C. (1999). Fluorimetric determination of niacin in foods by high-performance liquid chromatography with post-column derivatization. *Food Chemistry*, 65(1), 129-133.

Satir, G., & Guzel-Seydim, Z. B. (2016). How kefir fermentation can affect product composition?. *Small Ruminant Research*, 134, 1-7.

**S1B.Calibration curves**

**HPLC calibration curve and equations for water-soluble vitamins**

**
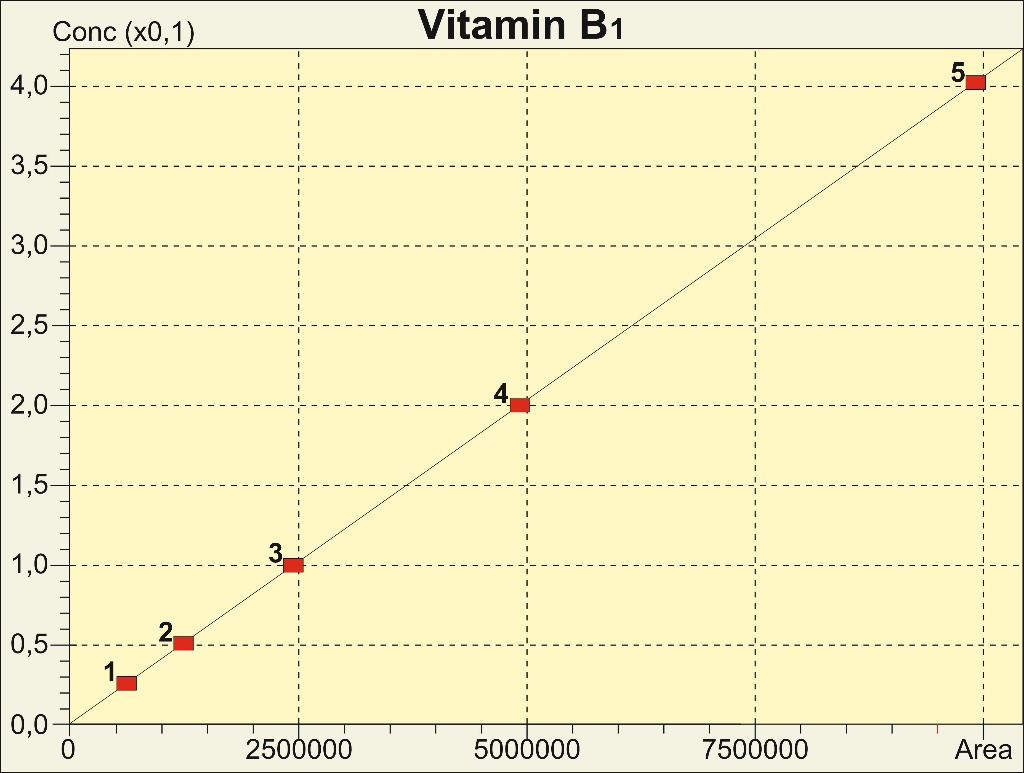
**

Y = aX + b; a = 4.069965e-008, b = 5.449319e-004, R^2^ = 0.99

**
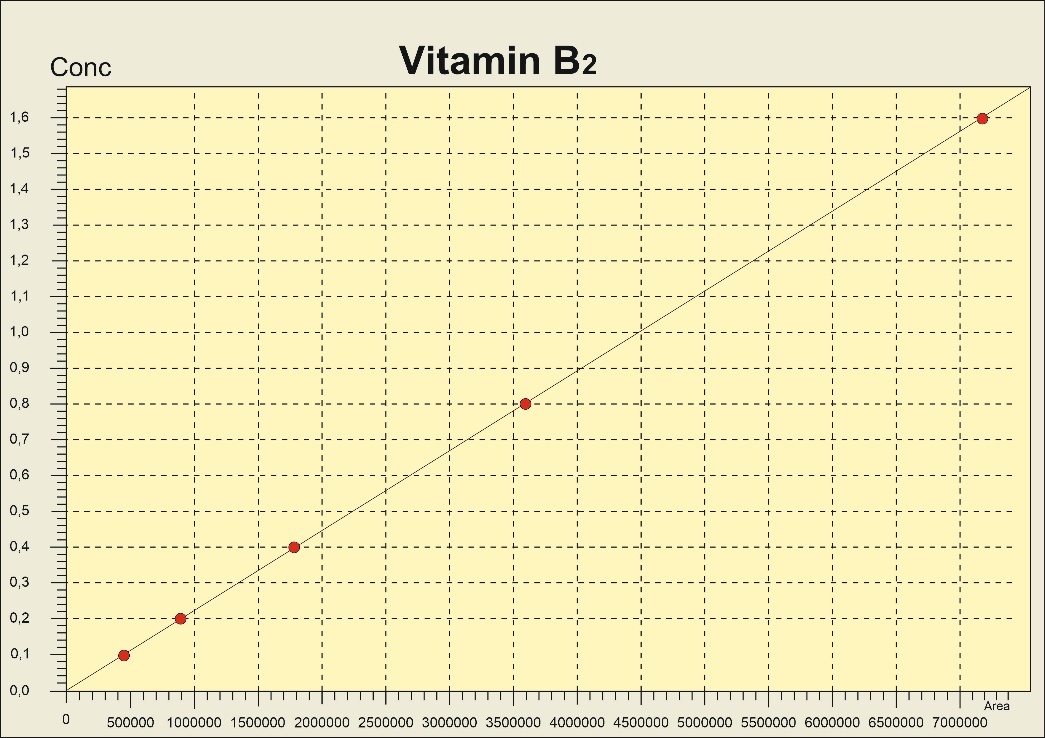
**

Y = aX + b; a = 2.2091e-007, b = -1.221197e-003, R^2^ = 0.99

**
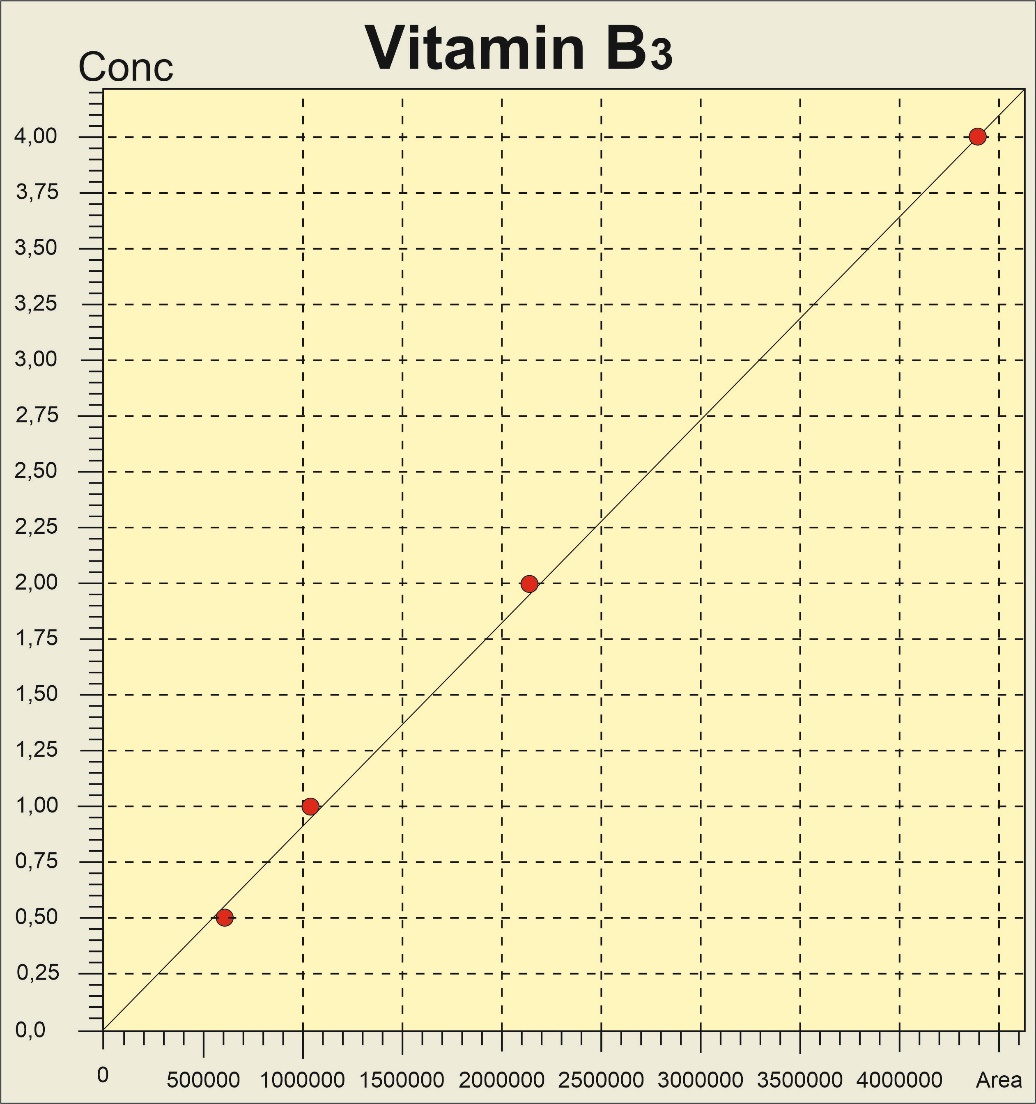
**

Y = aX + b; a = 9.137032e-007, b = 5.814605e-003, R^2^ = 0.99

**
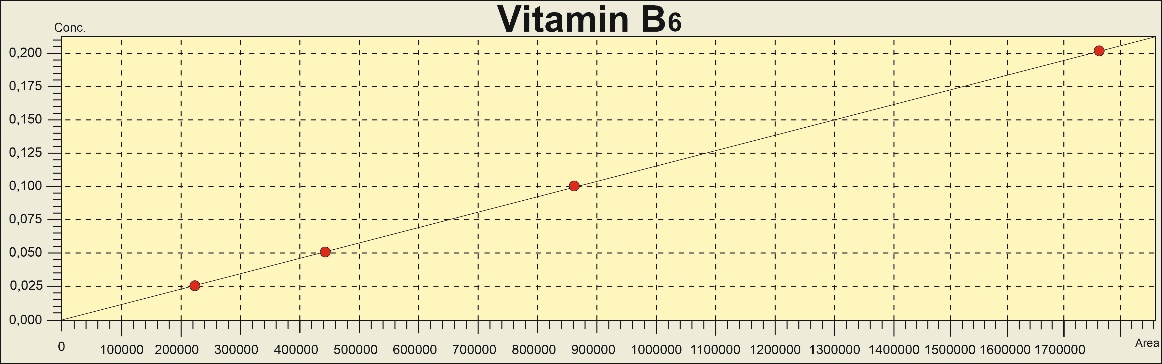
**

Y = aX + b; a=1.127511e-007, b = -2.091941e-004, R^2^ = 0.99

**
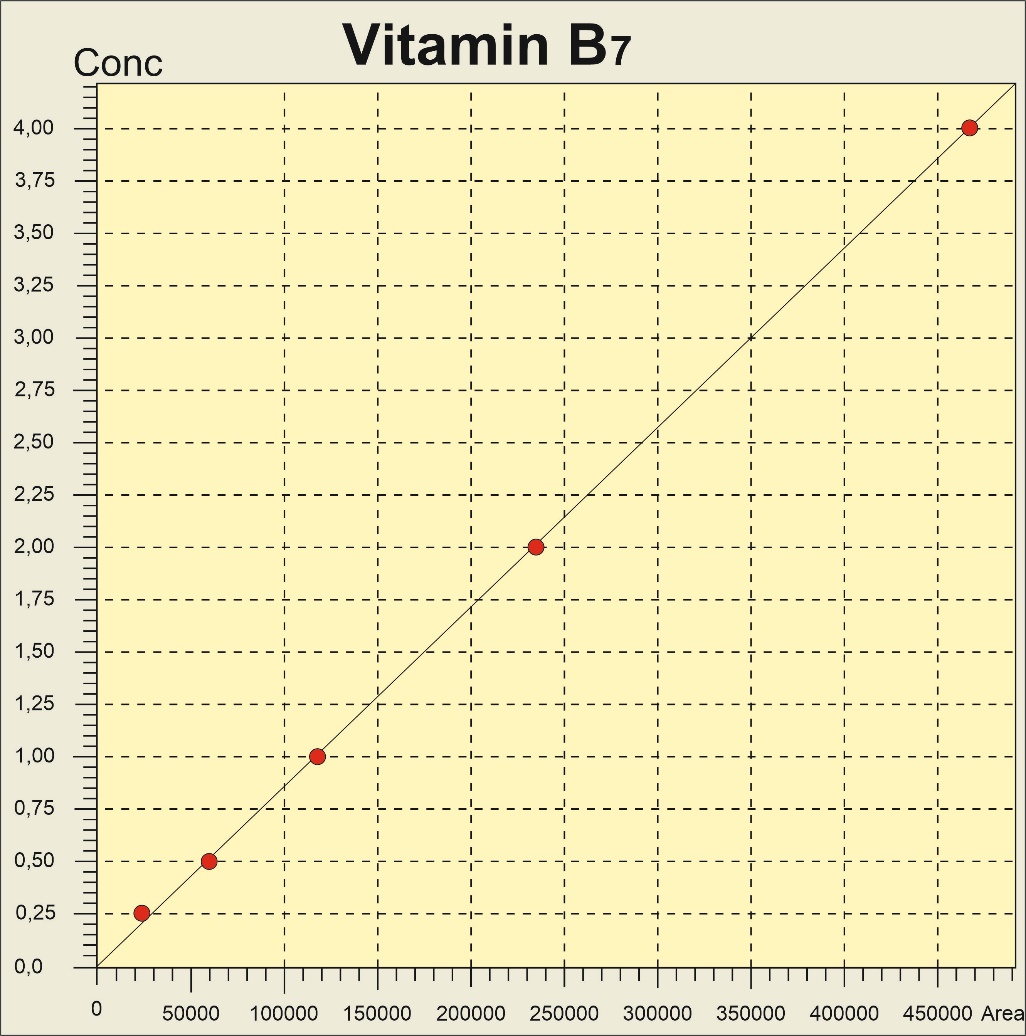
**

Y = aX + b; a = 8.686663e-006, b = -5.520184e-002, R^2^ = 0.99

**
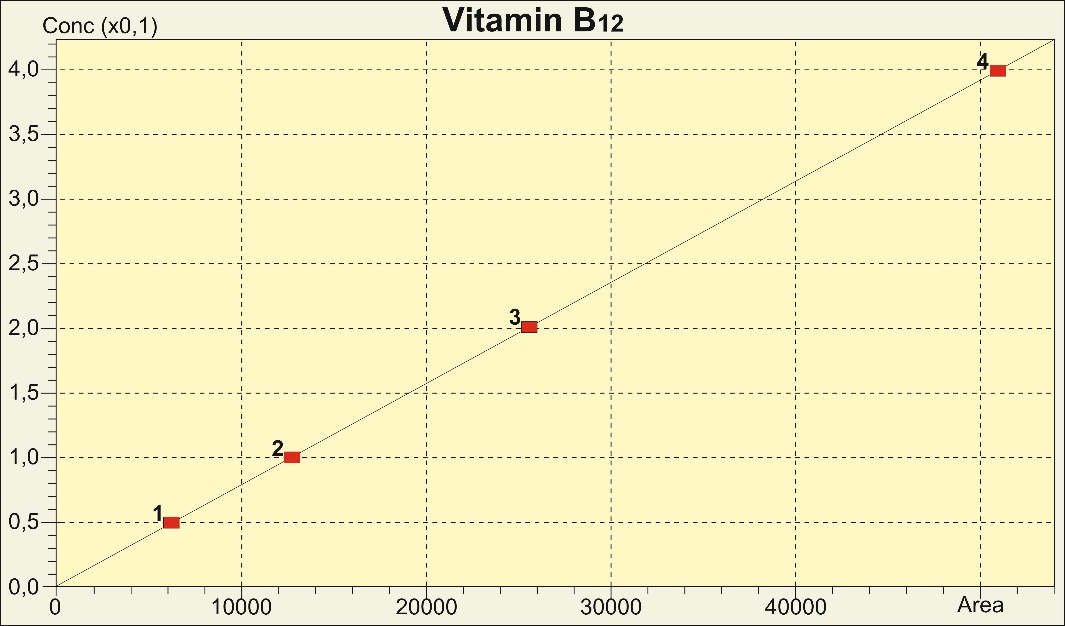
**

Y = aX + b; a = 7.788956e-006, b = 4.98816e-004, R^2^ = 0.99

**
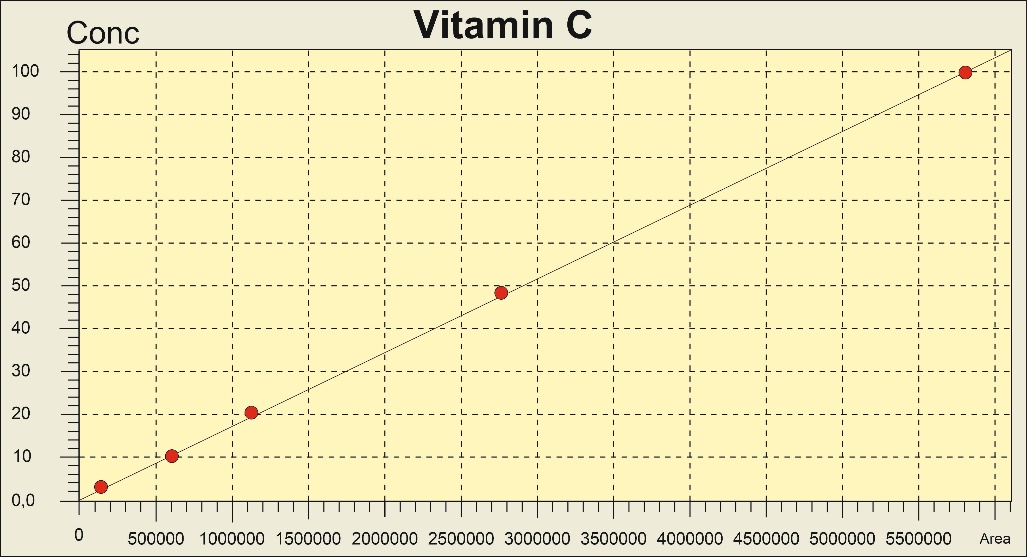
**

Y = aX + b; a = 1.735431e-005, b = 0.2997988, R^2^ = 0.99

Table S1. Running conditions of inductively coupled plasma optical emission spectrometer

OES measurements.

| Name of parameter | Value of parameter |
| --- | --- |
| PlasmaGasFlow  AuxiliaryGasFlow  NebulizerGasFlow  Power  TorchCassettePosition  PumpSpeed  Purge  Resolution  Integration Time  Read Delay  Replicates | 15 L/min  0.2 L/min  0.6 L/min  1450 Watts  -3  1.5 mL/minute  Normal  Normal  10 secondsmin/20 secondsmax  60 seconds  3 |

Table S2. HPLC operating conditions for water-soluble vitamins analysis

| **Vitamins** | **HPLC** | **Column** | **Detector** | **Mobile phase** | **Flow rate** | **Injection volume** | **Gradient profile** |
| --- | --- | --- | --- | --- | --- | --- | --- |
| **B_1_** | Shimadzu, RF-20A | Agilent ZORBAX  Eclipse XDB-C-18  (4.6×150 mm, 5 µm) | Fluorescence  Detector  (Ex: 366 nm, Em: 445 nm) | 10 mM KH_2_PO_4_  (pH 7.0-7.1)  Methanol: Water  25/75 (v/v) | 0.8 ml/min | 20 µL | Isocratic elution |
| **B_2_** | Shimadzu, RF-20A | Agilent ZORBAX  Eclipse XDB-C-18  (4.6×150 mm, 5 µm) | Fluorescence detector  (Ex: 445 nm, Em:530 nm) | Methanol:Water  25/75 (v/v) | 1.0 ml/min | 20 µL | Isocratic elution |
| **B_3_** | Shimadzu, RF-20A | Agilent LiChrosper 60  RP-select B  (250 mm×4.0 mm, 5 µm) | Fluorescence detector  (Ex: 325 nm, Em:480 nm) | 50 mM KH_2_PO_4_  % 30 H_2_0_2_: 7.5 ml  Cu_2_SO_4_ sol: 1ml  1000 ml water | 1.0 ml/min | 20 µL | Isocratic elution |
| **B_6_** | Shimadzu, RF-20A | ACE 5 C18  (250 mm×4.6 mm, 5 µm) | Fluorescence detector  (Ex: 290 nm, Em:395 nm) | 50 mM KH_2_PO_4_  (pH 2.8-2.9)  Water: Acetonitrile  95/5(v/v) | 1.0 ml/min | 50 µL | Isocratic elution |
| **B_7_** | Shimadzu, LC-20AD | ACE 5 C18  (250 mm×4.6 mm, 5 µm) | UV detector  (200 nm) | Water: Acetonitrile  875/125  250 µl trifluoro acetic acid (v/v) | 0.8 ml/min | 100 µL | Isocratic elution |
| **B_12_** | Shimadzu, LC-20AD | ACE 5 C18  (250 mm×4.6 mm, 5 µm) | UV detector  (361nm) | Water: Acetonitrile  875/125 (v/v)  250 µl trifluoroacetic acid | 0.8 ml/min | 100 µL | Isocratic elution |
| **C** | Shimadzu, LC-20AD | ACE 5 C18  (250 mm×4.6 mm, 5 µm) | UV detector  (254 nm) | 10 mM KH_2_PO_4_  (pH 2.8-2.9)  water 1000ml | 0.5 ml/min | 20 µL | Isocratic elution |
